# Supplementary material for: Coupling of melanocyte signaling and mechanics by caveolae is required for human skin pigmentation
Source: Nat Commun. 2020 Jun 12;11:2988. doi: 10.1038/s41467-020-16738-z (PMC7293304; doi:10.1038/s41467-020-16738-z)
Supplement: Supplementary file 1 — Supplementary Information [file 41467_2020_16738_MOESM1_ESM.pdf]

Supplementary Information

Coupling of melanocyte signaling and mechanics by caveolae is required for human skin pigmentation.

Lia Domingues, Ilse Hurbain, Floriane Gilles-Marsens, Julia Sirés-Campos, Nathalie André, Melissa Dewulf, Maryse Romao, Christine Viaris de Lesegno, Anne-Sophie Macé, Cédric Blouin, Christelle Guéré, Katell Vié, Graça Raposo, Christophe Lamaze, and Cédric Delevoye.

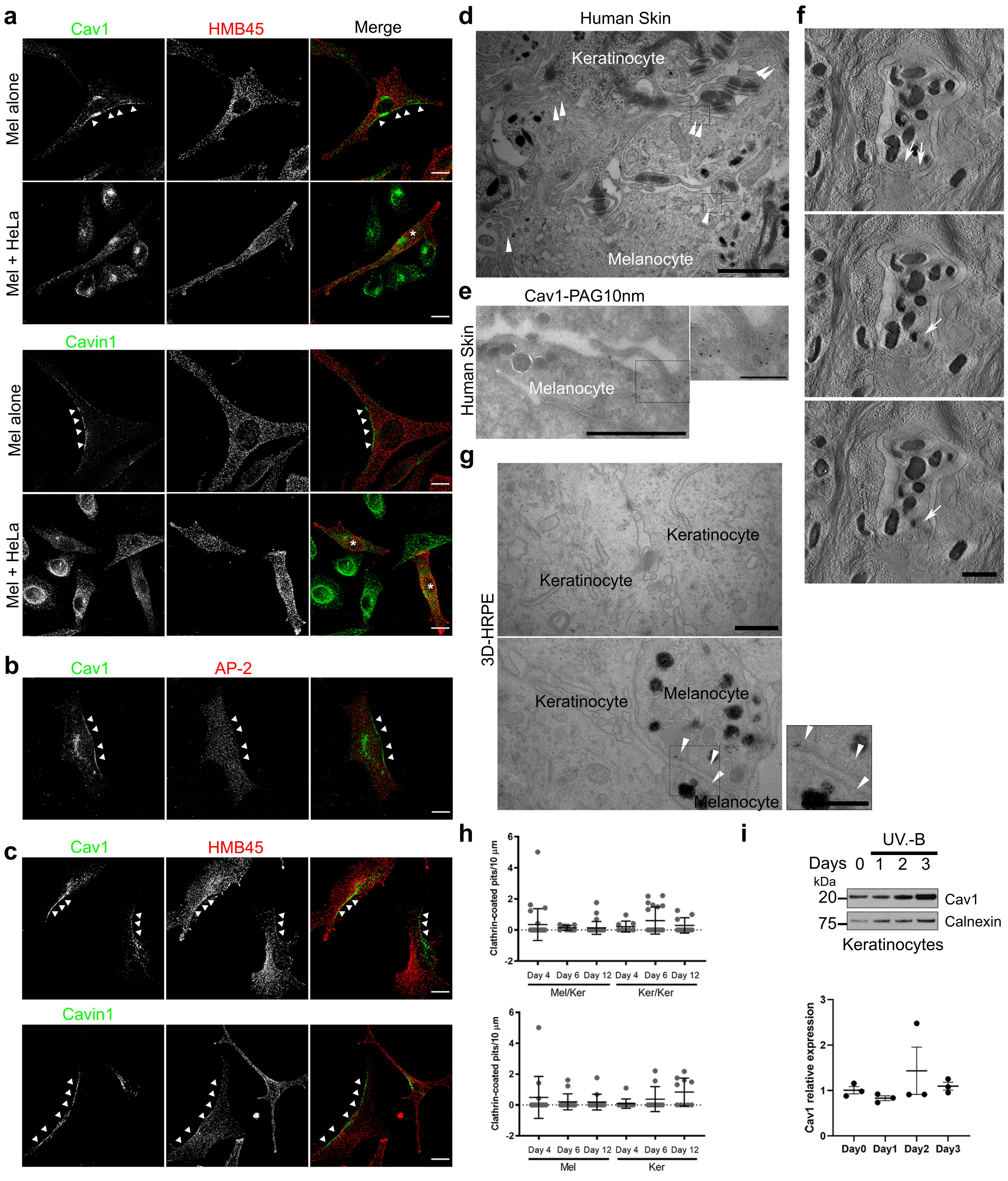
a
Cav1
HMB45
Merge
Mel alone
Mel + HeLa
Cavin1
b
Cav1
AP-2
c
Cav1
HMB45
Cavin1
d
Human Skin
Keratinocyte
Melanocyte
e
Cav1-PAG10nm
Human Skin
Melanocyte
f
g
3D-HRPE
Keratinocyte
Keratinocyte
Keratinocyte
Melanocyte
Melanocyte
h
Clathrin-coated pits/10 µm
Day 4
Day 6
Day 12
Mel/Ker
Ker/Ker
Mel
Ker
i
UV.-B
Days 0 1 2 3
kDa
20
75
Cav1
Calnexin
Keratinocytes
Cav1 relative expression
Day0
Day1
Day2
Day3

Supplementary Fig. 1 – Caveolae and clathrin coated pits localization and modulation in human epidermis and melanocytes cell culture systems.

a. IFM images of melanocytes (Mel, asterisks) in mono- or in co-culture with HeLa cells, fixed and immunolabelled for Cav1 (caveolin-1) or Cavin1 (top or bottom, respectively; green) and melanin (HMB45, red). **b**. IFM image of a melanocyte immunolabelled for Cav1 (green) and AP-2 (adaptor complex protein AP-2, red). **c**. IFM images of melanocytes grown in Ker-CM and immunolabelled for Cav1 or Cavin1 (top or bottom, respectively; green) and melanin (HMB45, red). (a-c) Arrowheads point Cav1 and Cavin1 polarization. Bars, 10 µm. **d**. Raw EM micrograph of human skin epidermis chemically fixed as represented in Fig. 1e. Arrowheads point plasma membrane invaginations with morphological features of caveolae. The boxed regions mark the area zoomed in the insets in Fig. 1e. Bar, 1 µm. **e**. Ultrathin cryosection of human skin epidermis immunogold labelled for Cav1 (PAG10nm). The boxed region marks the area zoomed in the inset. Bars: original, 1 µm; zoom, 250 nm. **f**. Slices of the electron tomographic reconstruction depicting the Mel-Ker interface shown in Fig. 1f. Large electron dense (black) structures correspond to melanin and arrows point plasma membrane invaginations resembling caveolae. Bar, 1 µm. See also **Supplementary Video 1**. **g**. Conventional EM micrographs of 3D-HRPE at day 6 showing keratinocyte-keratinocyte (top) or melanocyte-keratinocyte (bottom) interfaces. Arrowheads point melanocytes plasma membrane invaginations with morphological features of caveolae. The boxed region marks the area zoomed in the inset. Bars: original, 1 µm; zoom 0.5 µm. **h**. Number of CCP profiles per 10 µm of plasma membrane at the indicated interfaces (top) or cell types at melanocyte-keratinocyte interface (bottom) (for the number of interfaces and cells see Supplementary Table 1, g. and h.). **i**. Immunoblot analysis and quantification (n=3 independent experiments) of Cav1 expression levels in keratinocytes daily exposed to UV-B radiations (ultra-violet-B, 11 mJ/cm^2, 3 days). Quantification done relative to loading control. h and i, data are presented as mean ± s.e.m. h, comparison between interface/cells at the same time point: two-tailed unpaired t-test with Welch's correction; comparison between time points from the same cell type: one-way ANOVA with Tukey's multiple-comparison test; i, one-way ANOVA with Sidak's multiple comparison test.

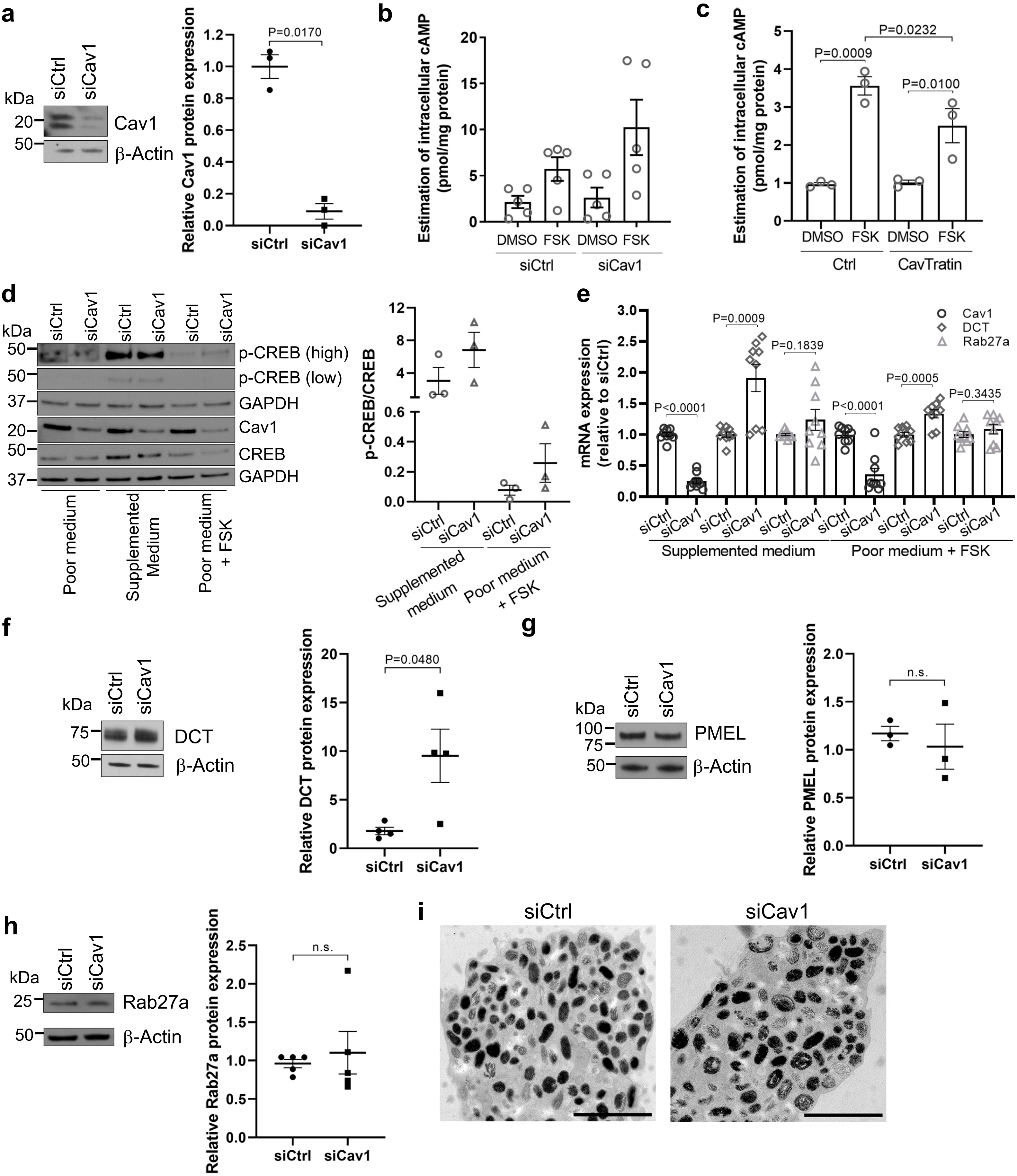
a
kDa
siCtrl
siCav1
20
50
Cav1
β-Actin
Relative Cav1 protein expression
P=0.0170
1.2
1.0
0.8
0.6
0.4
0.2
0.0
siCtrl
siCav1
b
Estimation of intracellular cAMP (pmol/mg protein)
20
15
10
5
0
DMSO
FSK
DMSO
FSK
siCtrl
siCav1
c
Estimation of intracellular cAMP (pmol/mg protein)
P=0.0232
P=0.0009
P=0.0100
5
4
3
2
1
0
DMSO
FSK
DMSO
FSK
Ctrl
CavTratin
d
kDa
siCtrl
siCav1
siCtrl
siCav1
siCtrl
siCav1
50
50
37
20
50
37
p-CREB (high)
p-CREB (low)
GAPDH
Cav1
CREB
GAPDH
Poor medium
Supplemented Medium
Poor medium + FSK
p-CREB/CREB
12
8
4
0.6
0.4
0.2
0.0
siCtrl
siCav1
siCtrl
siCav1
Supplemented medium
Poor medium + FSK
e
mRNA expression (relative to siCtrl)
3.0
2.5
2.0
1.5
1.0
0.5
0.0
P=0.0009
P=0.1839
P<0.0001
P<0.0001
P=0.0005
P=0.3435
Cav1
DCT
Rab27a
siCtrl
siCav1
siCtrl
siCav1
siCtrl
siCav1
siCtrl
siCav1
siCtrl
siCav1
siCtrl
siCav1
Supplemented medium
Poor medium + FSK
f
kDa
siCtrl
siCav1
75
50
DCT
β-Actin
Relative DCT protein expression
P=0.0480
20
15
10
5
0
siCtrl
siCav1
g
kDa
siCtrl
siCav1
100
75
50
PMEL
β-Actin
Relative PMEL protein expression
n.s.
2.0
1.5
1.0
0.5
0.0
siCtrl
siCav1
h
kDa
siCtrl
siCav1
25
50
Rab27a
β-Actin
Relative Rab27a protein expression
n.s.
2.5
2.0
1.5
1.0
0.5
0.0
siCtrl
siCav1
i
siCtrl
siCav1

Supplementary Fig. 2 – Depletion of caveolin-1 in stimulated melanocytes increases cAMP production and mRNA and protein expression levels of pigmentary-associated genes.

a. Immunoblot analysis of Cav1 expression levels in melanocytes treated with Ctrl (control) or caveolin-1 (Cav1) siRNA for 24h (left) and associated quantification. **b**. Quantification of cAMP intracellular concentration in melanocytes treated with siCtrl or siCav1 and incubated with DMSO (dimethyl sulfoxide) or 30 µM of FSK (forskolin) for 3h. **c**. Quantification of intracellular cAMP fold-change in melanocytes treated with Ctrl and CavTratin (Cav1 scaffolding domain, CSD) peptides for 7h and incubated with DMSO or 30 µM of FSK for 1h. **d**. Immunoblot analysis of p-CREB (phosphorylated cAMP responsive element binding protein) and CREB levels (left) in siCtrl- and siCav1-treated melanocytes maintained in poor medium, supplemented medium or in poor medium + FSK (15 min). GAPDH (Glyceraldehyde 3-phosphate dehydrogenase), loading control. Quantification of CREB activation (right), corresponding to the ratio of p-CREB on CREB total level after normalization with GAPDH. **e**. Quantification of Cav1, DCT (dopachrome tautomerase) and Rab27a mRNA expression levels in siCtrl- or siCav1-treated cells maintained in supplemented media or poor media + FSK for 3h and normalized to GAPDH expression levels. **f-h**. Immunoblot analysis of melanocytes treated for 5 days with siCtrl or siCav1 using the indicated antibodies (left) and associated quantifications (PMEL, using the antibody Pep13H, g). **i**. Conventional EM images representative of each condition. Bars: 2 µm. Quantifications of protein expression levels were done relative to the loading control. Values are the mean ± s.e.m. a, d and f-h, two-tailed paired t-test; b, one-way ANOVA with Holm-Sidak's multiple comparison test; c, one-way ANOVA with Holm-Sidak's multiple comparison test; e, two-tailed unpaired t-test with Welch's correction. See also **Supplementary Table 6**.

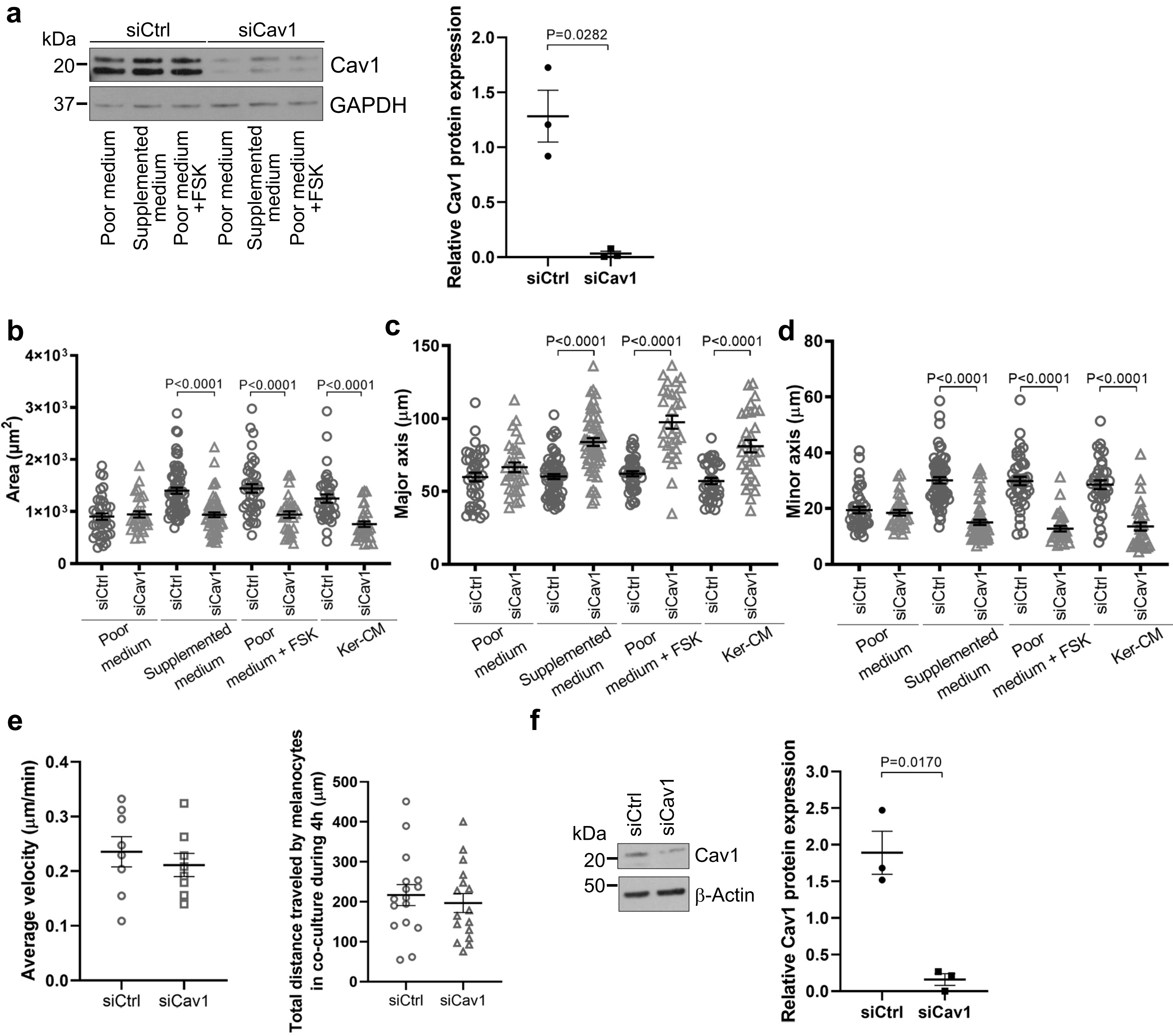
a
kDa
siCtrl
siCav1
20
37
Cav1
GAPDH
Poor medium
Supplemented medium
Poor medium +FSK
Poor medium
Supplemented medium
Poor medium +FSK
Relative Cav1 protein expression
P=0.0282
siCtrl
siCav1
b
Area (μm²)
P<0.0001
P<0.0001
P<0.0001
siCtrl
siCav1
Poor medium
Supplemented medium
Poor medium + FSK
Ker-CM
c
Major axis (μm)
P<0.0001
P<0.0001
P<0.0001
Poor medium
Supplemented medium
Poor medium + FSK
Ker-CM
d
Minor axis (μm)
P<0.0001
P<0.0001
P<0.0001
Poor medium
Supplemented medium
Poor medium + FSK
Ker-CM
e
Average velocity (μm/min)
siCtrl
siCav1
Total distance traveled by melanocytes in co-culture during 4h (μm)
siCtrl
siCav1
f
kDa
siCtrl
siCav1
20
50
Cav1
β-Actin
Relative Cav1 protein expression
P=0.0170
siCtrl
siCav1

Supplementary Fig. 3 – **Caveolae contributes to changes in melanocyte morphology but not their migration**.

a. Immunoblot analysis of Cav1 expression levels in melanocytes treated 48h with Ctrl (control) or Cav1 (caveolin-1) siRNA and maintained in the indicated media (left) and associated quantification (right; siCtrl: 1.2 ± 0.2, siCav1: 0.03 ± 0.02). **b**-**d**. Quantification of the area (b), major axis (c) and minor axis (d) of siCtrl- and siCav1-treated melanocytes grown in the conditions described in Fig. 3a (n=30 cells). **b**. siCtrl: Poor medium, 998.2 ± 68.4 - Supplemented medium, 1644 ± 73.2 - Poor medium + FSK (forskolin), 1501 ± 95.9 - Ker-CM (keratinocytes conditioned medium), 1251 ± 79.1; siCav1: Poor medium, 944.9 ± 61.0 - Supplemented medium, 1092 ± 64.3 - Poor medium + FSK, 941.4 ± 63 - Ker-CM, 758.8 ± 53.6. **c**. siCtrl: Poor medium, 64.6 ± 3.1 - Supplemented medium, 67.2 ± 2.5 - Poor medium + FSK, 61.6 ± 2.1 - Ker-CM, 57.2 ± 2.1; siCav1: Poor medium, 66.6 ± 3.3 - Supplemented medium, 87.4 ± 4.2 - Poor medium + FSK, 97.6 ± 4.5 - Ker-CM, 81.1 ± 4.3. **d**. siCtrl: Poor medium, 20.2 ± 1.4 - Supplemented medium, 32.0 ± 1.6 - Poor medium + FSK, 31.1 ± 1.6 - Ker-CM, 28.5 ± 1.5; siCav1: Poor medium, 18.5 ± 1.0 - Supplemented medium, 17.5 ± 1.5 - Poor medium + FSK, 12.8 ± 1.0 - Ker-CM, 15.6 ± 1.5. **e**. Quantification of the average velocity and total distance traveled by the melanocytes in co-culture with keratinocytes for a total of 4h as described in Fig. 3d and 3e (left, siCtrl:0.24 ± 0.03, siCav1:0.21 ± 0.02; right, siCtrl: 216.7 ± 26.3, siCav1:197.0 ± 23.7). **f**. Immunoblot analysis of Cav1 expression levels in melanocytes treated 72h with siCtrl and siCav1 (left) and associated quantification (right; siCtrl: 1.9 ± 0.3, siCav1: 0.16 ± 0.08). Quantifications of protein expression levels were done relative to the loading control. Values are the mean ± s.e.m. a-f, 3 independent experiments. a and f, two-tailed paired t-test; b-d, one-way ANOVA with Tukey's multiple comparison test, e, two-tailed unpaired t-test with Welch's correction.

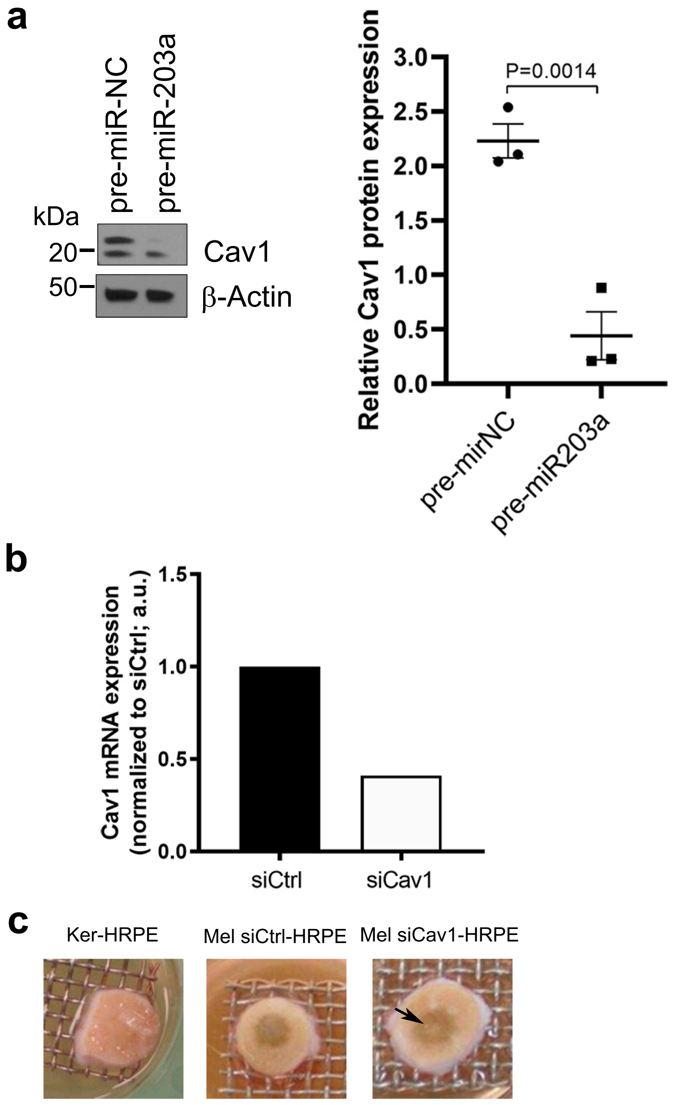
a
pre-miR-NC
pre-miR-203a
kDa
20
50
Cav1
β-Actin
Relative Cav1 protein expression
3.0
2.5
2.0
1.5
1.0
0.5
0.0
P=0.0014
pre-mirNC
pre-miR203a
b
Cav1 mRNA expression
(normalized to siCtrl; a.u.)
1.5
1.0
0.5
0.0
siCtrl
siCav1
c
Ker-HRPE
Mel siCtrl-HRPE
Mel siCav1-HRPE

Supplementary Fig. 4 – Efficiency of depletion of caveolin-1 after pre-miR-treatment and skin coloration in different 3D-HRPE set-ups.

a. Immunoblot analysis of Cav1 expression levels in melanocytes treated with pre-miR-NC (pre-miRNA-negative control) or pre-miR-203a (pre-miRNA-203a) for 5 days (left) and associated quantification (right; pre-miR-NC: 2.2 ± 0.16, pre-miR-203a: 0.44 ± 0.22; n=3 independent experiments). Values are the mean ± s.e.m. **b**. Cav1 mRNA levels in melanocytes treated 9 days with siCtrl or siCav1 were analyzed by quantitative qPCR (siCav1: 0.41). **c**. Macroscopic images of 3D-HRPE (human reconstructed pigmented epidermis) reconstructed with keratinocytes alone (left, Ker-HRPE), keratinocytes and siCtrl-treated melanocytes (middle, Mel siCtrl-HRPE) or keratinocytes and siCav1-treated melanocytes (right, Mel siCav1-HRPE). Arrow points towards the de-pigmented area. a, two-tailed paired t-test.

Supplementary Tables

Supplementary Table 1: Data relative to Figure 1.

Panel	Condition	Data	Number (n)/ experiment
b.	Cav1	Box 1: 61.8 ± 5.7; Box 2: 12.9 ± 2.5	12 cells
	Cavin1	Box 1: 58.7 ± 3.9; Box 2: 15.9 ± 2.5	
c.	Mono-culture	Cav1: 30.7 ± 3.5%; Cavin1: 30.7 ± 2.4%	150 cells, 3 independent experiments
	Co-culture Ker	Cav1: 54.7 ± 5.7%; Cavin1: 49.3 ± 3.5%	
	Co-culture HeLa	Cav1: 26.0 ± 5.0%; Cavin1: 28.0 ± 2.3%	
d.	Mel supplemented medium	Cav1: 20.0 ± 2.3%; Cavin1: 14.7 ± 3.0%	150 cells, 3 independent experiments
	Ker medium	Cav1: 8.0 ± 1.6%; Cavin1: 7.3 ± 3.6%	
	Ker-CM	Cav1: 46.7 ± 1.8%; Cavin1: 47.7 ± 1.3%	
g.	Mel-Ker	Day 4: 2.9 ± 0.7	28 interfaces
		Day 6: 3.4 ± 0.7	26 interfaces
		Day 12: 3.6 ± 0.6	20 interfaces
	Ker-Ker	Day 4: 0.7 ± 0.2	13 interfaces
		Day 6: 1.1 ± 0.3	9 interfaces
		Day 12: 0.9 ± 0.3	11 interfaces
h.	Mel	Day 4: 1.2 ± 0.4	59 cells
		Day 6: 5.0 ± 1.0	64 cells
		Day 12: 4.1 ± 0.9	60 cells
	Ker	Day 4: 4.5 ± 1.1	88 cells
		Day 6: 1.7 ± 0.8	117 cells
		Day 12: 3.1 ± 0.7	99 cells
g., h.	3D-HRPE normalized melanin content (a.u.): day-4, 1; day-6, 2.4		
i.	Day 0	Cav1: 0.5 ± 0.1	3 independent experiments
	Day 1	Cav1: 0.3 ± 0.1	
	Day 2	Cav1: 2.3 ± 0.7	
	Day 3	Cav1: 3.5 ± 1.0	

Supplementary Table 2: Data relative to Figure 2.

Panel	Condition	Data	Number (n) /experiment
a.	siCtrl	FSK: 3.2 ± 0.5	5 independent experiments
	siCav1	FSK: 6.6 ± 1.3	
b.	CavTratin:	0.66 ± 0.08	3 independent experiments
c.	Supplemented medium	siCtrl: 1.0 ± 0.1	3 replicates; 3 independent experiments
		siCav1: 1.3 ± 0.1	

	Poor medium + FSK	siCtrl: 1.0 ± 0.1	
		siCav1: 1.4 ± 0.1	
**d.**	Tyr	siCtrl: 2.3 ± 0.4	4 independent experiments
		siCav1: 3.1 ± 0.3	
	Cav1	siCtrl: 2.8 ± 0.5	
		siCav1: 0.3 ± 0.1	
**e.**	siCav1:	1.5 ± 0.2	4 independent experiments
**g.**	Stage I	siCtrl: 1.3 ± 0.5	siCtrl: StageI, n=20; Stage II, n=194; Stage III, n=787; Stage IV, n=285; total of 1286 melanosomes. siCav1: Stage I, n=19; Stage II, n=147; Stage III, n=1158; Stage IV, n=609; total of 1933 melanosomes. 19 cells each condition; 4 independent experiments
		siCav1: 1.1 ± 0.3	
	Stage II	siCtrl: 14.0 ± 2.0	
		siCav1: 7.9 ± 1.6	
	Stage III	siCtrl: 64.5 ± 5.6	
		siCav1: 58.0 ± 5.2	
	Stage IV	siCtrl: 20.1 ± 5.6	
		siCav1: 33.0 ± 5.8	

Supplementary Table 3: Data relative to Figure 3.

Panel	Condition	Data	Number (n) /experiment
**b.** (>2 prot.)	Poor medium	siCtrl: 38.0 ± 4.2	150 cells; 3 independent experiments
		siCav1: 21.7 ± 3.7	
	Supplemented medium	siCtrl: 65.7 ± 3.4	
		siCav1: 39.5 ± 3.9	
	Poor medium + FSK	siCtrl: 82.0 ± 4.0	
		siCav1: 46.0 ± 4.2	
	Ker-CM	siCtrl: 56.7 ± 0.7	
		siCav1: 26.7 ± 1.8	
**c.**	Poor medium	siCtrl: 3.5 ± 0.3	Poor medium: siCtrl and siCav1, n=30 cells. Supplemented medium: siCtrl, n=61 cells; siCav1, n=57 cells. Poor medium + FSK: siCtrl and siCav1, n=30 cells. Ker-CM: siCtrl, n= 37 cells; siCav1, n=31 cells; 3-6 independent experiments
		siCav1: 4.0 ± 0.3	
	Supplemented medium	siCtrl: 2.2 ± 0.1	
		siCav1: 6.8 ± 0.4	
	Poor medium + FSK	siCtrl: 2.3 ± 0.2	
		siCav1: 8.9 ± 0.7	
	Ker-CM	siCtrl: 2.5 ± 0.3	
		siCav1: 8.9 ± 1.1	
**e.**	No contact	siCtrl: 4.5 ± 1.3	siCtrl, n=182; siCav1, n=210 contacts; 3 independent experiments
		siCav1: 7.5 ± 1.4	
	Up to 1h	siCtrl: 53.1 ± 11.1	

		siCav1: 70.2 ± 6.2	
	From 1 to 4h	siCtrl: 44.1 ± 9.6	
		siCav1: 26.4 ± 3.9	
g.	siCtrl	Poor medium: 0.5 ± 0.2	6 independent experiments
		Supplemented medium: 2.2 ± 0.4	
		Poor medium + FSK: 1.5 ± 0.08	
	siCav1	Poor medium: 1.0 ± 0.3	
		Supplemented medium: 0.6 ± 0.2	
		Poor medium + FSK: 0.5 ± 0.1	
i.	siCtrl	50.0 ± 2.0	siCtrl, n= 714 cells; siCav1, n= 958 cells; 3 independent experiments
	siCav1	74.4 ± 1.5	

Supplementary Table 4: Data relative to Figure 4.

Panel	Condition	Data	Number of cells/ experiment
b.	siCtrl	71.9 ± 5.7	150 cells; siCtrl and siCav1, 4 independent experiments, pre-miR-NC and pre-miR-203a, 3 independent experiments
	siCav1	48.5 ± 7.0	
	Pre-miR-NC	81.5 ± 3.7	
	Pre-miR-203a	48.6 ± 8.3	
c.	siCtrl	0.1 ± 0.02	siCtrl, n=98; siCav1, n=93, 4 independent experiments; pre-miR-NC, n=111; pre-miR-203a, n=93; 3 independent experiments
	siCav1	0.03 ± 0.004	
	Pre-miR-NC	0.1 ± 0.01	
	Pre-miR-203a	0.04 ± 0.006	
e.	Mel siCtrl	5.7 ± 1.8	siCtrl, n= 21; siCav1, n=15; 1 experiment
	Mel siCav1	1.7 ± 0.7	

Supplementary Table 5: Sequences of primers used for the qPCR.

Primers		Sequence (5'→3')
hCav1	Fw	GCGACCCTAAACACCTCAAC
	Rev	ATGCCGTCAAAACTGTGTGTC
hTYR	Fw	TGCACAGAGAGACGACTCTTG
	Rev	GAGCTGATGGTATGCTTTGCTAA
hDCT	Fw	AACTGCGAGCGGAAGAAACC
	Rev	CGTAGTCGGGGTGTACTCTCT
hRab27a	Fw	GCTTTGGGAGACTCTGGTGTA
	Rev	TCAATGCCCACTGTTGTGATAAA
hGAPDH	Fw	CTGGGCTACACTGAGCACC
	Rev	AAGTGGTCGTTGAGGGCAATG

Supplementary Table 6: Data relative to Supplementary Fig.2.

Panel	Condition	Data	Number of cells/ experiment
**a.**	siCtrl	1.0 ± 0.07	3 independent experiments
	siCav1	0.09 ± 0.05	
**b.**	siCtrl	DMSO: 2.1 ± 0.7	5 independent experiments
		FSK: 5.7 ± 1.3	
	siCav1	DMSO: 2.6 ± 1.1	
		FSK: 10.2 ± 3.0	
**c.**	Ctrl + FSK	3.7 ± 0.4	3 independent experiments
	CavTratin + FSK	2.5 ± 0.5	
**d.**	Supplemented medium	siCtrl: 3.0 ± 1.6	3 independent experiments
		siCav1: 6.8 ± 2.2	
	Poor medium + FSK	siCtrl: 0.08 ± 0.03	
		siCav1: 0.26 ± 0.13	
**e.**	Supplemented medium Cav1	siCtrl: 1.0 ± 0.03	3 replicates; 3 independent experiments
		siCav1: 0.25 ± 0.04	
	Supplemented medium DCT	siCtrl: 1.0 ± 0.03	
		siCav1: 1.7 ± 0.2	
	Supplemented medium Rab27a	siCtrl: 1.0 ± 0.02	
		siCav1: 1.2 ± 0.2	
	Poor medium + FSK Cav1	siCtrl: 1.0 ± 0.04	
		siCav1: 0.36 ± 0.1	
	Poor medium + FSK DCT	siCtrl: 1.0 ± 0.03	
		siCav1: 1.3 ± 0.07	
	Poor medium + FSK Rab27a	siCtrl: 1.0 ± 0.05	
		siCav1: 1.1 ± 0.08	
**f.**	DCT	siCtrl: 1.7 ± 0.4	4 independent experiments
		siCav1: 9.5 ± 2.8	
**g.**	PMEL	siCtrl: 1.2 ± 0.08	5 independent experiments
		siCav1: 1.0 ± 0.23	
**h.**	Rab27a	siCtrl: 0.96 ± 0.05	5 independent experiments
		siCav1: 1.1 ± 0.3	
