# Supplementary material for: Coupling of melanocyte signaling and mechanics by caveolae is required for human skin pigmentation
Source: Nat Commun. 2020 Jun 12;11:2988. doi: 10.1038/s41467-020-16738-z (PMC7293304; doi:10.1038/s41467-020-16738-z)
Supplement: Supplementary file 3 — Description of Additional Supplementary Files [file 41467_2020_16738_MOESM3_ESM.pdf]

Description of Additional Supplementary Files

File Name: Supplementary Movie 1
Description: Ultrastructural 3D-model of a Melanocyte-Keratinocyte interface at a human skin epidermis by electron tomography; melanocytes plasma membrane (green), keratinocytes plasma membrane (blue), pigmented melanosomes (red), melanin pigment (black) and caveolae (white) in single and clustered structures. See also **Fig. 1f** and **Supplementary Fig. 1f**.

File Name: Supplementary Movie 2
Description: Time-lapse microscopy of siCtrl-treated melanocytes co-cultured with keratinocytes used to draw the cell boundaries (see also **Fig. 3d**, top). Trans-illumination. Acquisition parameter: 200 ms exposure. Video is shown at 7 frames/second. Bar, 20 µm.

File Name: Supplementary Movie 3
Description: Time-lapse microscopy of siCav1-treated melanocytes co-cultured with keratinocytes used to draw cell boundaries (see also **Fig. 3d**, bottom). Trans-illumination. Acquisition parameter: 200 ms exposure. Video is shown at 7 frames/second. Bar, 20 µm.

File Name: Supplementary Movie 4
Description: Time-lapse microscopy of siCtrl-treated melanocytes (contoured in yellow, left) co-cultured with keratinocytes (contoured in green, left). See also **Fig. 3e**. Trans-illumination. Acquisition parameter: 200 ms exposure. Video is shown at 7 frames/second. Bar, 25 µm.

File Name: Supplementary Movie 5
Description: Time-lapse microscopy of siCav1-treated melanocytes (contoured in yellow) co-cultured with keratinocytes (contoured in green). See also **Fig. 3e**. Trans-illumination. Acquisition parameter: 200 ms exposure. Video is shown at 7 frames/second. Bar, 25 µm.

File Name: Supplementary Movie 6
Description: Time-lapse microscopy of the burst assay for siCtrl melanocytes. See also **Fig. 3h**. Acquisition parameters: 80-150 ms. Video is shown at 7 frames/second. Bar, 50µm.

File Name: Supplementary Movie 7
Description: Time-lapse microscopy of the burst assay for siCav1melanocytes. See also **Fig. 3h**. Acquisition parameters: 80-150 ms. Video is shown at 7 frames/second. Bar, 50µm.

File Name: Supplementary Code 1
Description: README file detailing the procedure to use the two codes developed in the study. The codes are provided as .ijm files that can be opened using the ImageJ software.
