# Supplementary material for: Coupling of melanocyte signaling and mechanics by caveolae is required for human skin pigmentation
Source: Nat Commun. 2020 Jun 12;11:2988. doi: 10.1038/s41467-020-16738-z (PMC7293304; doi:10.1038/s41467-020-16738-z)
Supplement: Supplementary file 4 — Supplementary Code 1 [file 41467_2020_16738_MOESM4_ESM.zip › Supplementary Macro/readMe_FILE.pdf]

Requirements:

- Fiji
  - Ilastik plugin (available in the Fiji Updater)
  - "Shape Smoothing" plugin of Fiji, available here https://imagej.net/Shape_Smoothing
- Ilastik

Tested on: Windows 7 and 10, with Fiji 1.52p & Ilastik 1.3.2

General workflow:

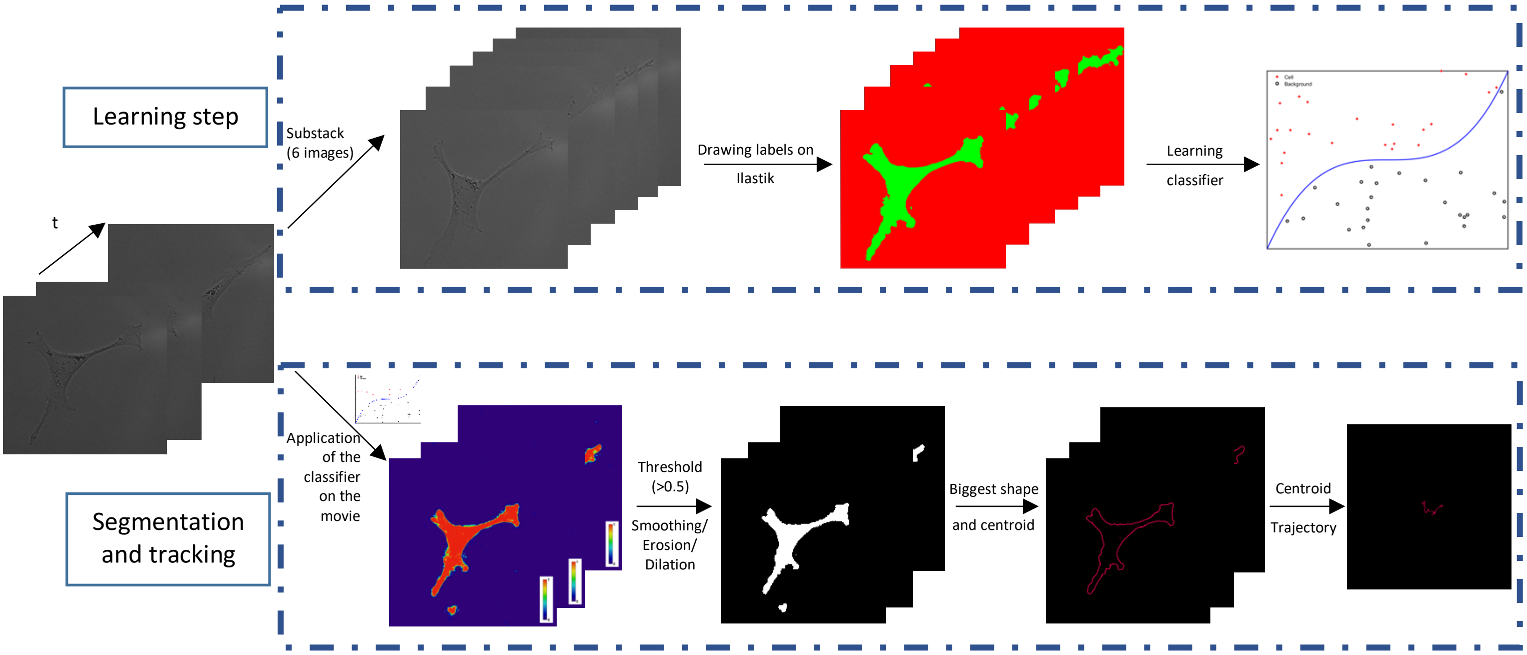

1/ Macro1_segmentation.ijm

- Ask the user for a file to analyze
- Ask for the user for a folder of Results
- Pre-treat the data (subtract background)
- Save a sub-stack for Ilastik, made of 6 frames, equally distributed in time names as the file chosen + "_smallStack"
- Save the whole stack pre-processed as the file chosen + "_AllStack"
- Save a copy of the original data

2/ Pixel Classification in Ilastik

- Open Ilastik
- Choose « Pixel classification »

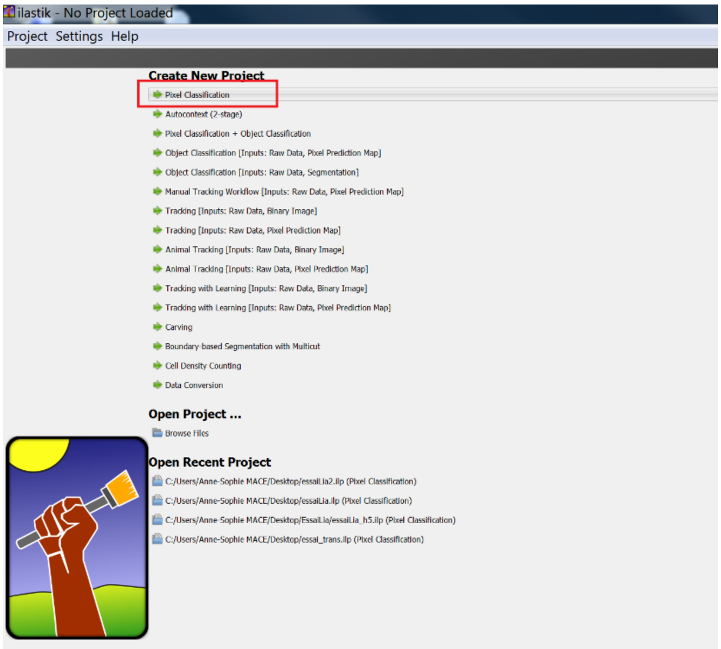

If possible, save the project in the same folder as the images.

- Choose “Add new Image” -> “Add Separate Images” and take the “_smallStack” (less images -> faster)

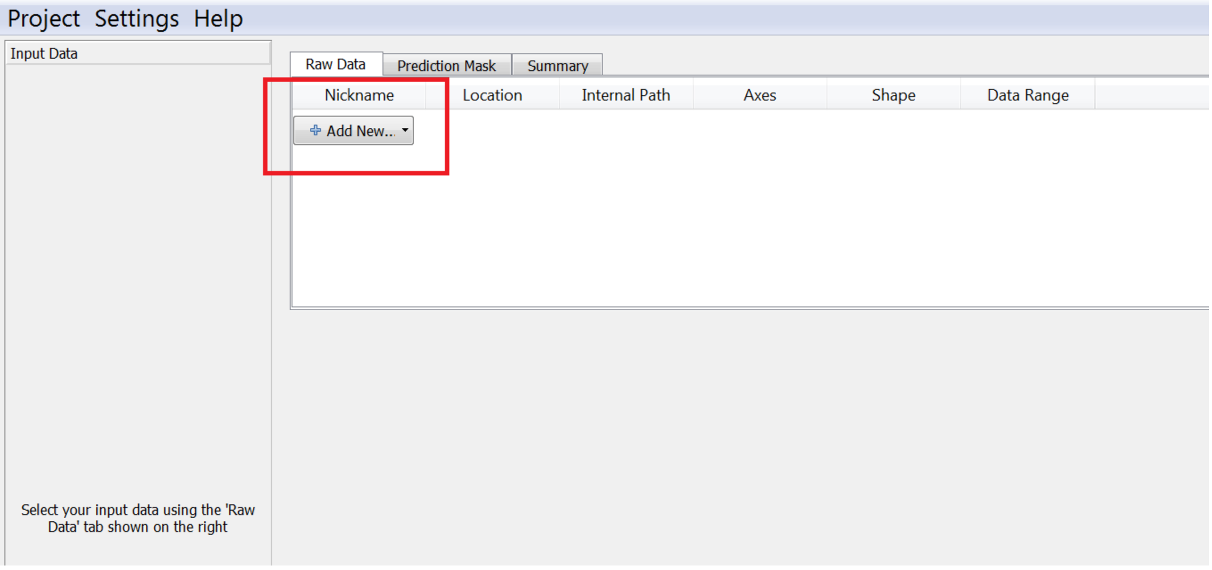

- Feature selection: Select Features, Choose them all

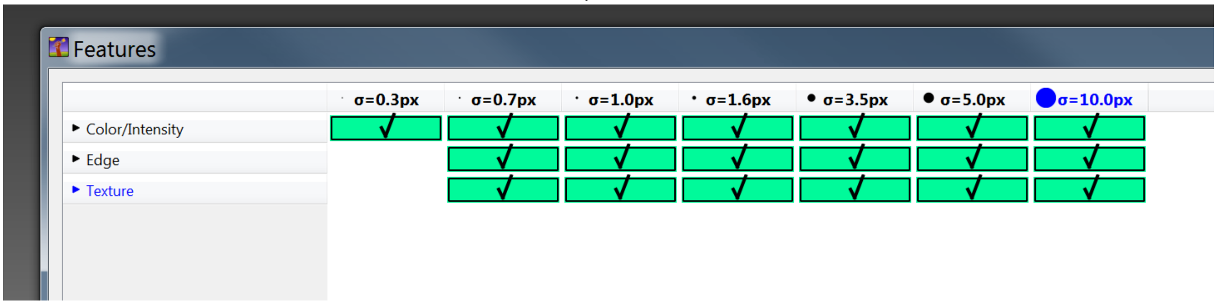

- Training: add two labels, start with the background then the cell (the order is not important in Ilastik, but it will be when treating the results in ImageJ) and draw areas for both; do not hesitate to use a big size for the background; when you finish click on “Live Update” (can be long); compare the results in the different images and if needed continue with the annotation process.

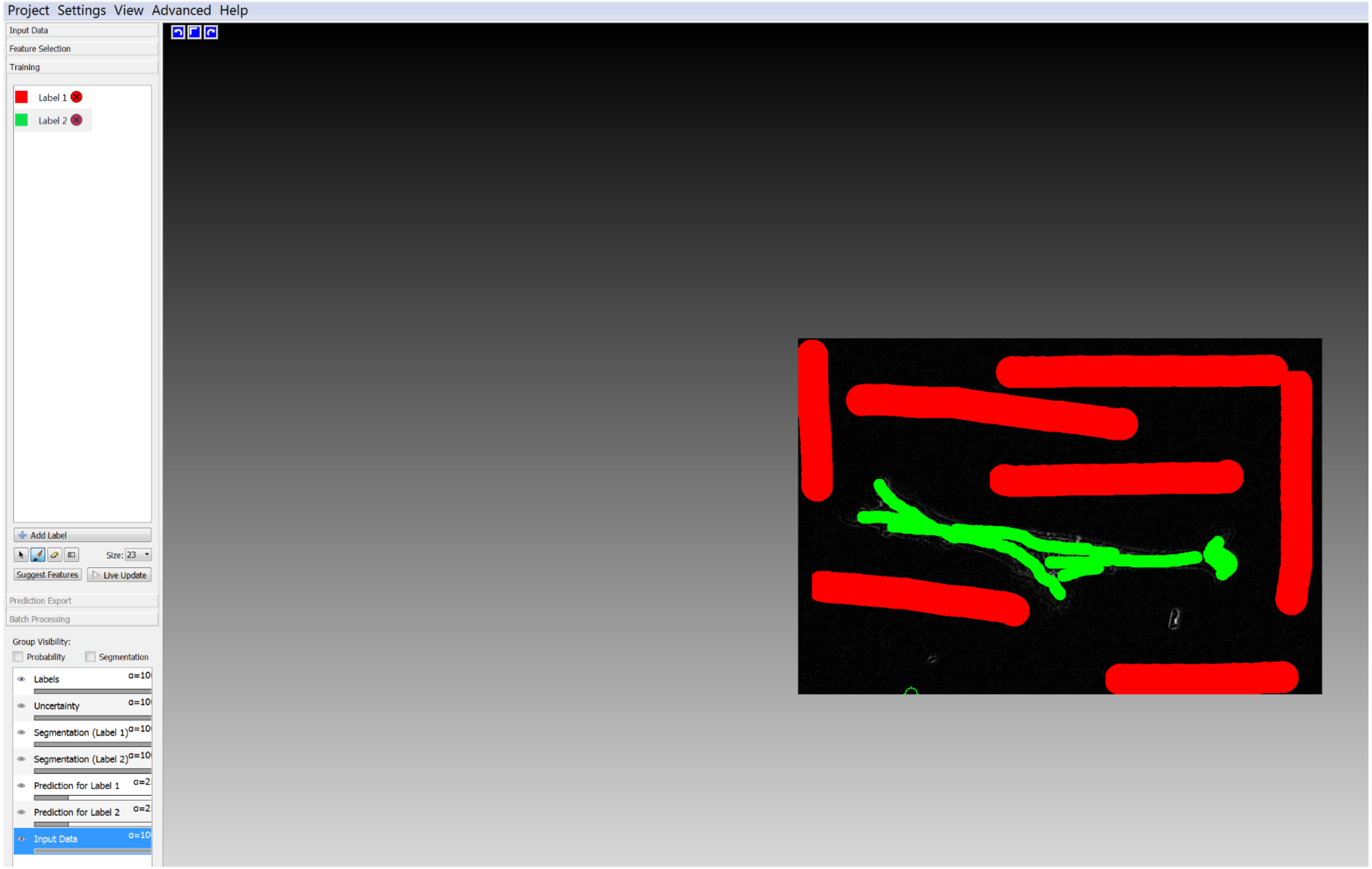

- When you are satisfied with the result on each image, click on “Batch processing” followed by “Select Raw Data Files”, select your file “AllStack” and then click on “Process all files” (can be long depending on the size of the stack).

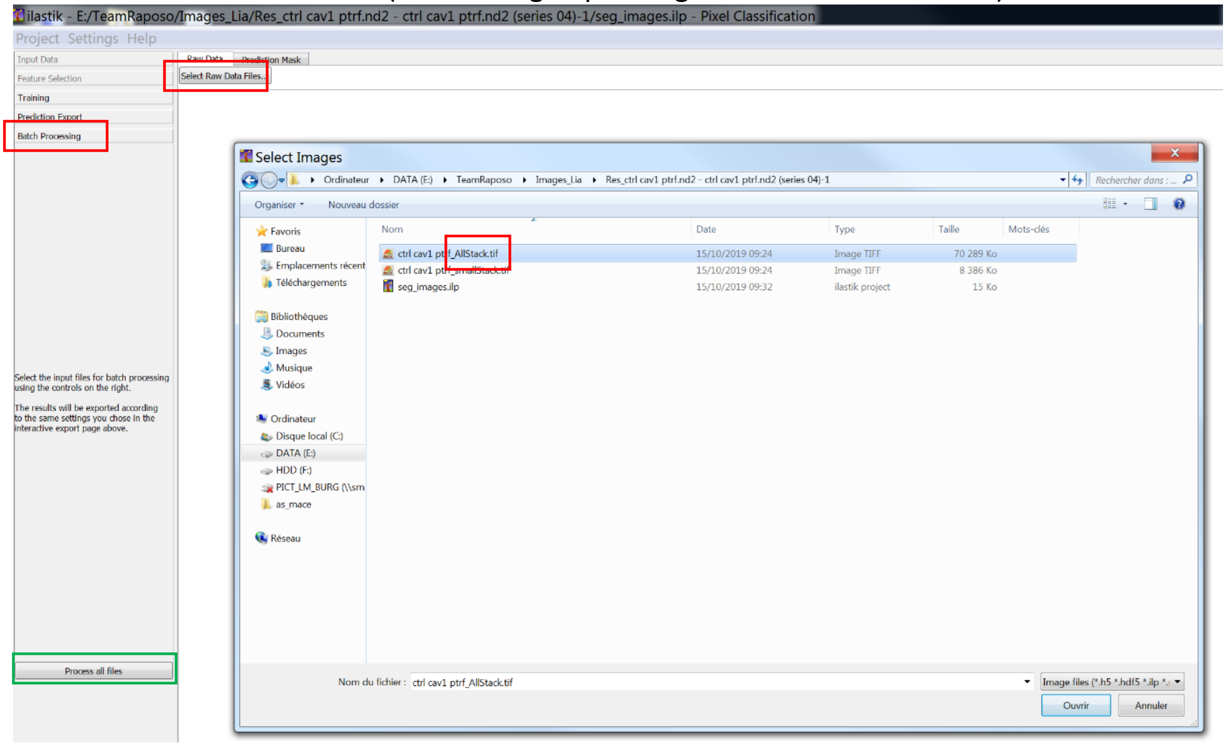

When finished, a file *.h5 with the same name as the AllStack image will be created.

<u>3/ Macro2_res_ilastik.ijm</u>

- Asks the user for a probability map (created in Ilastik)
- Reads the H5 file and creates the mask by thresholding at 0.5
- Smooths slightly the shapes
- For each frame keeps only one Region of Interest, the biggest one
- Asks the user for pixel size (seems to be lost during Ilastik treatment) and time frame (for the speed computation)
- Shows all ROIs to the user with the possibility of manual correction
  ➔ *In this case, the wrong ones were removed, and the “Interpolate ROIs” tool of the ROI Manager was used to replace them.*
- Creates a movie of the shapes (masks) evolving
- Creates an image of the whole trajectory
- Saves the result table containing for each frame: area/shape descriptor/centroid and speed (distance divided by frame time)
- Saves the final ROIs
- If the original image is in the same folder, the macro displays the overlay of the masks on this image
