# Supplementary material for: Coupling of melanocyte signaling and mechanics by caveolae is required for human skin pigmentation
Source: Nat Commun. 2020 Jun 12;11:2988. doi: 10.1038/s41467-020-16738-z (PMC7293304; doi:10.1038/s41467-020-16738-z)
Supplement: Supplementary file 12 — Reporting Summary [file 41467_2020_16738_MOESM12_ESM.pdf]

nature research

Corresponding author(s): Cedric Delevoye and Lia Domingues

Last updated by author(s): Apr 23, 2020

Reporting Summary

Nature Research wishes to improve the reproducibility of the work that we publish. This form provides structure for consistency and transparency in reporting. For further information on Nature Research policies, seeAuthors & Referees and theEditorial Policy Checklist .

Statistics

For all statistical analyses, confirm that the following items are present in the figure legend, table legend, main text, or Methods section.

n/a	Confirmed	
	x	The exact sample size (*n*) for each experimental group/condition, given as a discrete number and unit of measurement
	x	A statement on whether measurements were taken from distinct samples or whether the same sample was measured repeatedly
	x	The statistical test(s) used AND whether they are one- or two-sided<br>*Only common tests should be described solely by name; describe more complex techniques in the Methods section.*
x		A description of all covariates tested
x		A description of any assumptions or corrections, such as tests of normality and adjustment for multiple comparisons
	x	A full description of the statistical parameters including central tendency (e.g. means) or other basic estimates (e.g. regression coefficient) AND variation (e.g. standard deviation) or associated estimates of uncertainty (e.g. confidence intervals)
	x	For null hypothesis testing, the test statistic (e.g. *F*, *t*, *r*) with confidence intervals, effect sizes, degrees of freedom and *P* value noted<br>*Give P values as exact values whenever suitable.*
x		For Bayesian analysis, information on the choice of priors and Markov chain Monte Carlo settings
x		For hierarchical and complex designs, identification of the appropriate level for tests and full reporting of outcomes
x		Estimates of effect sizes (e.g. Cohen's *d*, Pearson's *r*), indicating how they were calculated

Our web collection on statistics for biologists contains articles on many of the points above.

Software and code

Policy information about availability of computer code

Data collection	Metamorph (v.7.8.0.0), Nikon Imaging Software (v.4.0; NIS Elements), Applied Precision Deltavision CORE (V.4.1.0), ITEM (v.5.2; EMSIS)
Data analysis	ImageJ (v.1.52.s) for WB and IF images analysis. ITEM software (v.5.2; EMSIS) for EM analsyis. IMOD (v.4.9) for 3D EM reconstruction. GraphPad Prism (v.8.3) for statistics. Ilastik (v.2.0) for cell migration.

For manuscripts utilizing custom algorithms or software that are central to the research but not yet described in published literature, software must be made available to editors/reviewers. We strongly encourage code deposition in a community repository (e.g. GitHub). See the Nature Research guidelines for submitting code & software for further information.

Data

Policy information about availability of data

All manuscripts must include a data availability statement. This statement should provide the following information, where applicable:

- Accession codes, unique identifiers, or web links for publicly available datasets
- A list of figures that have associated raw data
- A description of any restrictions on data availability

Authors can confirm that all relevant data are included in the paper and/ or its supplementary information files. All other relevant data are provided in the “source data file" (Fig1b,c,d,g,h,i; Fig2a,b,c,d,e,g; Fig3b,c,e,f,g,i; Fig4b,c,e; FigS1h,i; FigS2a,b,c,d,e,f,g,h; FigS3a,b,c,e,f; FigS4a,b)

Field-specific reporting

Please select the one below that is the best fit for your research. If you are not sure, read the appropriate sections before making your selection.

Life sciences study design

All studies must disclose on these points even when the disclosure is negative.

Sample size	No sample-size pre-calculations were made. Sample size was based on the reproducibility between independent experiments and using as reference the 95% confidence interval. For immunoblots, melanin quantification, cAMP measurements, RT-PCR, immunofluorescence and time-lapse imaging, all experiments were carried out at least 3 times independently, including the usage of different donors, cells treatment, recovering samples and processing. The number of caveolae/um of PM analysis followed Hurbain et al, 2017 (DOI:10.1016/j.jid.2017.09.039). The bursting assay followed Dewulf et al, 2019 (DOI:10.1038/s41467-019-09405-5). The melanin transfer followed Ripoll et al, 2018 (DOI:10.1083/jcb.201709055).
Data exclusions	No data was excluded.
Replication	All replicates are reported in the manuscript.
Randomization	The data was not randomized. Primary cells came from different donors and samples were grouped by siRNA-/miRNA-/peptide-treatment.
Blinding	Investigators were not blinded. Different donors of cells were used in the experiments and the cells responsiveness to treatments needed to be verified in control cells. Tissue analysis was done by two different investigators.

Reporting for specific materials, systems and methods

We require information from authors about some types of materials, experimental systems and methods used in many studies. Here, indicate whether each material, system or method listed is relevant to your study. If you are not sure if a list item applies to your research, read the appropriate section before selecting a response.

Materials & experimental systems

n/a	Involved in the study	
☐	☒	Antibodies
☐	☒	Eukaryotic cell lines
☒	☐	Palaeontology
☒	☐	Animals and other organisms
☐	☒	Human research participants
☒	☐	Clinical data

Methods

n/a	Involved in the study	
☒	☐	ChIP-seq
☒	☐	Flow cytometry
☒	☐	MRI-based neuroimaging

Antibodies

Antibodies used	rabbit anti-Caveolin1 (clone 2297; BD Transduction Laboratories; 1:5000 [IB]; 1:200 [IFM]); rabbit anti-PTRF (Cavin-1; Abcam; 1:200 [IFM]); mouse anti-HMB45 (recognizing PMEL-positive fibrils onto which melanin deposits, used here as a 'melanin marker'; clone HMB45; abcam; 1:200 [IFM]); mouse anti-α adaptin (clone AP6; abcam; 1:50 [IFM]); mouse anti-Tyrosinase (clone T311; Santa Cruz biotechnology; 1:200 [IB]); mouse anti-DCT (clone C-9; Santa Cruz biotechnology; 1:200 [IB]); rabbit anti-Pep13h (Raposo et al., 2001; 1:200 [IB]); goat anti-Rab27a (SICGEN; 1:1000 [IB]); rabbit anti-phosphorylated CREB (Ser133) (clone 87G3; Cell Signaling technology; 1:1000 [IB]); rabbit anti-CREB (clone 48H2; Cell Signaling Technology; 1:1000 [IB]); mouse anti-phosphorylated MLC2 (Ser19) (#3675; Cell Signaling Technology; 1:1000 [IB]); rabbit anti-MLC2 (clone D18E2; Cell Signaling Technology; 1:1000 [IB]); mouse anti-ACTB (β-actin; clone AC-74; Sigma; 1:2000 [IB]); rabbit anti-GAPDH (clone GAPDH-71.1; Sigma; 1:10000 [IB]); rabbit anti-Calnexin (ADI-SPA-860; Enzo Life Sciences; 1:1000 [IB]). Secondary antibodies coupled to horseradish peroxidase (HRP) were used at 1:10000 (ab6721, ab6789, ab97057, [IB], Abcam). Secondary antibodies and phalloidin conjugated to 488, 555 and 647 Alexa dyes were used at 1:200 ([IFM], Invitrogen).
Validation	We used commercial available antibodies except for Pep13h (available from Hearing's or Marks's lab and validated in previous studies (e.g. Berson et al., 2001; Harper et al., 2008; Kobayashi et al., 1994; Raposo et al., 2001)). All validations were done by the manufacturers or described in previous studies as follows:<br>-Cav1: https://www.bdbiosciences.com/eu/reagents/research/antibodies-buffers/cell-biology-reagents/cell-biology-antibodies/purified-mouse-anti-caveolin-1-2297caveolin-1/p/610407; Western blot (Routinely Tested); Immunohistochemistry, Immunoprecipitation, Immunofluorescence (Tested During Development).<br>-Cavin1 https://www.abcam.com/ptrf-antibody-ab48824.html: Suitable for: IP, IHC-P, ICC/IF, ELISA, WB.<br>-HMB45 https://www.abcam.com/melanoma-gp100-antibody-hmb45-ab787.html: Suitable for: Flow Cyt, IHC-P, ICC/IF.<br>-α adaptin: https://www.abcam.com/alpha-adaptin-antibody-ap6-ab2730.html Suitable for: ICC/IF, ICC, Inhibition Assay, Electron Microscopy, ELISA, Blocking, IP, WB, IHC-FoFr, Flow Cyt.<br>-Tyrosinase: https://www.scbt.com/p/tyrosinase-antibody-t311 Tyrosinase (T311) is recommended for detection of Tyrosinase of mouse, rat and human origin by Western Blotting.<br>-DCT: https://www.scbt.com/fr/p/trp2-antibody-c-9?requestFrom=search TRP2 (C-9) is recommended for detection of TRP2 of mouse, rat and human origin by Western Blotting.<br>-Rab27a: http://www.sicgen.pt/product/rab27a-polyclonal-antibody_1_32 Specificity: Detects Rab27a by Western blot in the following human, rat and mouse whole cell lysates and transfected cells with GFP-Rab27a. This antibody is specific for Rab27a. It

does not recognise Rab27b protein.
-p-CREB and CREB: DOI: 10.7554/eLife.54298.
-p-MLC: https://www.cellsignal.com/products/primary-antibodies/phospho-myosin-light-chain-2-ser19-mouse-mab/3675 Application: western blotting.
-MLC: https://www.cellsignal.com/products/primary-antibodies/myosin-light-chain-2-d18e2-rabbit-mab/8505 Application: Western Blottting.
-actin: https://www.sigmaaldrich.com/catalog/product/sigma/a2228?lang=fr&region=FR western blot: 0.5-1 µg/mL using total cell extracts of human or chicken fibroblasts.
-GAPDH: https://www.sigmaaldrich.com/catalog/product/sigma/g8795?lang=fr&region=FR western blot: 0.025-0.05 µg/mL using A431 total cell extract.
-Calnexin: https://www.enzolifesciences.com/ADI-SPA-860/calnexin-polyclonal-antibody/ Applications: ICC, IF, IHC (PS), IP, WB.

Eukaryotic cell lines

Policy information about cell lines

Cell line source(s)	HeLa Kyoto (obtained from the Bruno Goud's team, CNRS UMR144, Institut Curie, Paris, France)
Authentication	The cell line used in the manuscript was not authenticated, and used here as an irrelevant cell (negative control).
Mycoplasma contamination	The cell lines used in this study were regularly tested for mycoplasma contamination and tested negative.
Commonly misidentified lines (See ICLAC register)	The cell line used in the manuscript is not listed in the database of commonly misidentified cell lines maintained by ICLAC.

Human research participants

Policy information about studies involving human research participants

Population characteristics	Healthy women (mean age 29.6 +/- 7.9 yrs).
Recruitment	No participants were recruited. The skins analysed were obtained from surgical leftovers of residues of breast or abdominal reduction operations. No bias to report.
Ethics oversight	Written informed consent was obtained in accordance with the Helsinski Declaration and with article L.1243-4 of the French Public Health Code. Given its special nature, surgical residue is subject to specific legislation included in the French Code of Public Health (anonymity, gratuity, sanitary/safety rules…). This legislation does not require prior authorization by an ethics committee for sampling or use of surgical waste (http://www.ethique.sorbonne-paris- cite.fr/?q=node/1767).

Note that full information on the approval of the study protocol must also be provided in the manuscript.
